# Dr. Cuming, in Answer to Mr. Thomas

**Published:** 1806-03

**Authors:** Ralph Cuming

**Affiliations:** H. M. S. Pegasus, Harwich


### *To the Editors of the Medical and Physical Journal.*

GENTLEMEN,

THAT time must surely be mispent, which is employed in criticism for the purpose of gratifying a spirit of malevolence, which too frequently rankles in the breast of those, whose education and situation in civil society, should have taught them better motives; but liberal conduct and charitable sentiments are qualifications which are rarely met with in many medical disquisitions. I have to lament, in common with the generality of your readers, that many men are more prone to cavil at, and disparage the endeavours of their neighbours to promote medical knowledge, than to lend a helping hand themselves; such beings may properly be denominated, *les ronces et les epines de la société,* which so often spring up, and choke the growth of useful plants. But it must be remembered that it is much easier for a man to criticize the writings of others, than it is for him to write himself; because he has the the advantage of ideas, not his own, which he can twist and pervert, agreeably to the bent of his natural disposition. I shall be silent on the first part of Mr. Thomas's critique on my paper in the November Journal, as it relates to the Editors, and censures them for want of discrimination in their selection of papers. Impressed with a due sense of the propriety of the following sentence of an antient author, *Antefero iniquissimam pacem justissimo bello,* I could most willingly lay down my pen, did I not think it justifiable to defend myself against a violent and unjust attack, intended to sully my reputation; this circumstance will of itself be a sufficient apology for my intrusion on the patience of your readers, to give me an indulgent hearing, and for yourselves, in permitting me to "answer for myself," through the medium of your justly esteemed, though too frequently abused publication. The premier part of Mr. T's attack is chiefly confined to that portion of my paper, which related to the most expeditious method of making leeches bite; had he pointed out a better mode for the application of these useful animals, some degree of praise would have been due to him; but this would have been a task too difficult for him to perform. And should he have imagined that every medical tyro, had been already acquainted with the knowledge that I was inculcating, I believe he was grossly mistaken; for I do not think that there will be any vanity in the supposition, that I really conveyed a lesson which many of my medical brethren will consider extremely useful and beneficial, and which I really think he may have been ignorant of himself. I have fortunately been honoured with public testimonials of the utility of my communications from some of your correspondents, whose names and writings rank with those that do honor to the Medical and Physical Journal: Mr. Cooke of London, Mr. Dawson of Sunderland, and others, whose names I do not recollect, nor have I time at present to search for them, merit my esteem and warmest thanks. Other testimonials I have from private correspondence of physicians, not less grateful to my feelings.

A man of Mr. Thomas's manners should be reminded of, De quoque viro et cui dicas sæpe caveto. At the conclusion of his comments on the leeches, he observes, "Seriously, *Mr. Editors,* if all this had been treasured up, miser-like, in the rusty coffers of the Doctor's own soul, I do not think the improvement of your readers would have suffered any very considerable diminution."

Now

Now let me seriously advise Mr. T. when he turns critic again, not to jumble his jargon in such a manner with another person's words, as to leave no criterion whereby they may be distinguished from his own; for at treasured up, he places his inverted commas, but does not mark the end of his quotation.

I must, before quitting this subject, observe, that should any medical gentleman be acquainted with a better and more expeditious method of making leeches bite, than that which I have already pointed out, I shall be greatly obliged to him for the desirable information; yet I am well assured that many, if not Mr. Thomas, were entirely unacquainted with a method equal to that which I did myself the pleasure of recommending.

The gentleman now commences the most serious part of his critique, by prefacing it with "I will now take the liberty of proceeding to his case of Ischuria; the recital of it makes one shudder at his patient's narrow escape from imminent danger, but in which I cannot see that the Doctor has much reason to plume himself on his knowledge and dexterity."

Mr. C. the subject of my paper, was on a visit at the house of a friend, who was a married man, and motives of delicacy prevented him from complaining, until he was in extreme agony, therefore had I supposed that my statement of his case would have been so improperly handled, I should have been more circumspect in my diction, than to have said he had not passed any urine for *many hours*.

Having said that I was foiled in my attempt to draw off the urine, on the evening in which he complained, after the catheter passed with great ease apparently as far as the membranous part of the urethra, or to the prostate gland, he observes, "Now comes the mischief," and which he attributes to my using a steady, firm, and cautious force, for I felt the sensation of something giving way to the point of the instrument, impressing me with the idea that bands were formed across the passage from the exudation of coagulable lymph, or that the passage was nearly or totally obliterated by surrounding inflammation, and that my patient possessed more than ordinary fortitude, and begged me to persevere. I found the instrument advance suddenly for the space of half an inch, but on withdrawing the stillette, nothing but a few drops of blood followed. I now deemed it prudent to desist; on removing the catheter, about an ounce of urine followed, with hard straining.

The

The latter part of the sentence he has omitted in his quotation, for very obvious reasons, and which I will shortly prove; he has also been guilty of misquotation.

Relative to the force, which I have stated to be *steady, firm,* and *cautious,* he has the presumption to imagine that it was greater than common prudence would have dictated; nay, it must appear that it was not, if aught may be conjectured from my patient's feelings, who seemed conscious, from the manner in which I treated him, of my *great care* in not aggravating his present sufferings; for violent usage will (when a sensible part is inflamed) cause the most stout-hearted man to cry out. The sensation of something giving way to the point of the instrument, conveying an idea that bands were formed across the passage, is what every person has witnessed, who has been in the habit of using the catheter, and I suppose may be attributed to the reason assigned. The sudden advance of the instrument for the space of half an inch (as I supposed) is easily accounted for, from its having overcome the obstruction in this part of the urethra.

The harsh and uncharitable inference drawn from this, will be obvious by Mr. T's own words; for he says, "From the foregoing statement, does it not amount to more than a presumption that the catheter was pushed through the membranous part of the urethra, between the bladder and rectum, or into the prostate gland."

That the former was not the case is rendered very evident by the flow of urine which followed, on withdrawing the instrument; but he tells you of drops of blood only, and artfully leaves out the urine in this place. So therefore the most presumptive, nay, certain proof is, that the point of the instrument was in the natural passage; for had this not been the case, it were likely that there would have been no evacuation of urine, which followed the removal of the instrument. What I have already said will induce any impartial person to suppose, that the point of the catheter had reached beyond the membranous part of the urethra, and was really in the *natural* passage, for had it been lacerated, it were a thousand to one against my passing it into the vesica urinaria, the next day.

He now proceeds to the oval tub with warm water.— It is not my intention to waste much more of my time, which has lately been engaged by a medical work which I am still busy with; but I must remark, that Mr. T. sneers at a mode of introducing the catheter, which I have recommended, by taking advantage of a state of deliquium sm and relaxation whilst the patient is immersed in warm water, in an *oval tub*, which could not be done in a round one, as its shape would preclude the possibility of effecting it, at such a favourable moment. It grieves me to observe, that Mr. T. appears to be one of those passive unfeeling men, who would stand as it were with their hands tied behind their backs, when the catheter meets with resistance, rather than use those endeavours which are calculated to prevent that fatal catastrophe, a rupture or mortification of the bladder. I have seen eminent men foiled in their endeavours to draw the urine off, who never thought of the plan which I adopted with such great success. But Mr. Thomas, if one may judge from what he has written, knew this, and every kind of knowledge that has appeared in your instructive Journal; then, what treasures of still greater knowledge have we not to expect will burst forth upon the benighted medical world, when this *blazing meteor* shall again make his entrée into the medico chirurgical sign of your Journal. After the bleeding and depleting means have failed; what remains to be done, except the dreadful alternative of puncturing per anum, or plunging a trocar through the parietes of the abdomen above the symphisis pubis, or operating on the perinæum? The fatal consequences of which have been too frequently felt. —Then, are we not to open the natural passage by such attempts as prudence and the judgment of the medical attendant may suggest?

The father of my patient is a physician, and I hope this paper may catch his eye, and that he may be induced to offer his sentiments on the case.

Had that accident occurred which Mr. T. would wish the world to believe, is it probable, nay I will say *possible*, that my patient should have experienced so rapid a recovery, for on the third day he voided his urine plenoflumine; therefore, if performing an expeditious and successful cure can be considered as bearing testimony to the propriety of the means adopted, I have nothing to fear from the insinuations of Mr. Thomas.

I am, &c.

RALPH CUMING, M. D.

*H. M. S. Pegasus, Harwich, January* 8, 1806.

